# Stress and midlife women’s health

**DOI:** 10.1186/s40695-018-0034-1

**Published:** 2018-03-16

**Authors:** Lynnette Leidy Sievert, Nicole Jaff, Nancy Fugate Woods

**Affiliations:** 1 Department of Anthropology, UMass Amherst, Machmer Hall, 240 Hicks Way, Amherst, MA 01003-9278 USA; 2 Department of Chemical Pathology, University of the Witwatersrand, Johannesburg, South Africa; 3 School of Nursing, University of Washington, Seattle, WA USA

**Keywords:** Stress, Midlife, Allostatic load

## Abstract

Stress is ubiquitous in everyday life, and chronic stress can have negative consequences for health and social welfare. Although a growing body of research addresses the relationships between stress, health, and quality of life, there is a gap in the literature with regard to the effects of stress among women at midlife. The purpose of this commentary is to provide a brief history of stress research, including various methods for measuring stress; discuss the physiological effects of stress; and review relevant studies about women at midlife in order to identify unanswered questions about stress. This commentary also serves as an introduction to a thematic series on stress and women’s midlife health where stress is examined in relation to a wide range of symptom experiences, in the context of family and negative life events, as associated with women’s work, and correlated with the challenges of violence and discrimination. The goal of this commentary and thematic series is to extend the conversation about stress to include women at midlife, and to examine where we are, and where we are going, in order to direct future research and provide relevant care for this growing population.

## Background

Stress is ubiquitous in contemporary life and an anticipated part of everyday living. Stress-related disorders are a pervasive concern, with negative consequences for health and social welfare [1]. Despite a growing body of research showing general links between stress, health, and quality of life, there are limited data about the effects of stress among midlife women and the relationship between stress and health at midlife.

The study of stress has been guided by a variety of definitions and conceptual frameworks, creating a wide and diverse understanding of this complex topic.

“Stress” as a concept has a long history in health-related literature. Hans Selye’s [2] pioneering work on stress described “the non-specific response of the body to any demand made on it” (p.692), and revealed the physiological consequences of induced stress in animal models, yielding information about the general adaptation syndrome [3]. Studies focusing on the hypothalamic-pituitary-adrenal (HPA) axis and the autonomic nervous system have linked stress responses, when experienced chronically, to a variety of illnesses.

Other avenues of research explore the relationship between stressful life events and illness. Investigators such as Holmes and Rahe [4] created an inventory of challenging life events, including the death of a close family member, a jail term, and change in financial circumstances, to provide a standardized measure for the effect of these stressors and the ability to cope with them. In turn, the Dohrenwends’ [5] research explored life events that were deemed most stressful as a foundation for understanding their effects on health.

In addition to major life events, there are everyday stresses/stressors that women experience, such as an unexpected minor illness of a family member who requires care, dealing with traffic during a commute, or changing work hours. Although not evaluated as challenging, these have a cumulative effect and contribute to, or are associated with, the outcomes of major life events [6].

Anthropologists have broadened the range of events considered to be stressful. For example, among Turkana women in East Africa, stress reactivity was associated with the frequent movement of encampments, major herd losses, and livestock raiding [7]. Modernization has long been associated with increased levels of stress biomarkers in traditional communities undergoing social and economic change [8, 9].

In addition, recent studies among U.S. populations, such as African American women at midlife, document unique experiences of stressors related to racism and micro-aggressions in everyday life [10, 11]. Other research suggests that psychiatric disorders in South Africa, though under-reported, may be the result of stressors such as racism and political upheaval during the apartheid years as well as ongoing gender inequality, criminal violence, and significant poverty [12].

Lazarus introduced a transactional model of stress in which the person’s response to stressful events became part of the equation. The individual was understood to be in constant transaction with the environment, cognitively assessing (appraising/evaluating) the situation and deciding whether or not she had the necessary resources or coping skills to meet the demands placed upon her [13]. This body of research has revealed a variety of ways of coping, including ways of focusing on the problem, ways of focusing on the emotional response to the problem, and how to manage both.

In addition, ecological models of understanding stress and stress response have been proposed. For example, culture influences both the meaning of stressful events and the availability of coping resources [14–16]. Social factors, such as networks of support, may help buffer the individual from experiences of stressors [17] or, in the case of conflict-ridden relationships, may exacerbate other stressors.

Women’s physiological responses to stressors are of interest in understanding health effects. The HPA axis and autonomic nervous system both contribute to responses characterized as flight or fight. Over time, the delayed return to physiological pre-stress levels may contribute to chronic elevation or blunting of cortisol or catecholamines and subsequent physiological dysfunction. Indeed, over the years, early conceptualizations of stress have given rise to additional concepts related to associated health effects, such as the notion of allostatic load advanced by McEwen [18] which describes the cumulative physiological effects of repeated and chronic stress.

As definitions and conceptualizations of stress have changed across time, so too have measures of stress and stress outcomes. Physiological pathways of interest to researchers include the HPA axis, the autonomic nervous system, and the immune system. Physiological indicators of stress and stress responses include cortisol (now measured in saliva, urine, nails and hair), catecholamines [19], blood pressure [20], telomere attrition [21], Epstein-Barr virus antibodies, and C-reactive protein assayed from dried blood spots [22, 23]. Novel collection tools include sweat pads, automated microdialysis systems, and smartphone applications [24]. Subjective assessment tools include the measure of self-reported experiences, such as the Perceived Stress Scale [25].

McEwen’s model of allostatic load incorporates multiple indicators of primary and secondary mediators of stress effects on health. Primary mediators include norepinephrine, epinephrine, cortisol, and dihydroepianderosterone sulfate (DHEAS). Secondary mediators include diastolic and systolic blood pressure, cholesterol, glycated hemoglobin, and waist:hip ratio [26]. HPA axis activation has been linked to hypothalamic-pituitary-ovarian (HPO) axis function, with extreme stressors interrupting HPO regulation of the menstrual cycle, resulting in amenorrhea. For midlife women, stress-related alteration in HPO function early in the lifespan may be associated with an increased risk of hot flashes [27].

Despite advances in this field of research, we remain relatively uninformed about the unique experiences of midlife women. Given the recognition of midlife as a stage of the lifespan in which important transitions occur, it is surprising that little attention has been focused on understanding the consequences of stress and women’s health. What remains to be addressed in the women’s health literature is the identification of the types of life events that women experience as stressful and their effect on reproductive health and healthy aging. For example, researchers suggest that increased complaints of mood disorders during the menopausal transition may be due not only to hormonal fluctuations, but also to psychosocial stressors that are common at midlife, including employment challenges, aging parents, adult children with adult problems, changing body image, loss of fertility and its implications, and relationship and sexual difficulties [28, 29]. In addition, data show that stress may result in the greater intensity and prevalence of some menopausal symptoms [30, 31], while stressful life events, particularly in childhood, may lead to an earlier age at menopause [32, 33].

Very recently, investigators interested in stress have begun to focus on midlife women. For example, an Australian study of 181 midlife and older women revealed that those who experienced stressors that caused them to feel helpless or fear for their lives reported higher body mass index and more chronic illness than those without these stressors. Duration of exposure to these stressors was associated with higher depressive symptoms and greater sleep disturbances [34]. Promising efforts include analyses of data from the Study of Women's Health Across the Nation (SWAN), examining the relationship of race/ethnicity, socioeconomic status, and psychosocial mediators of allostatic load. For instance, analyses of longitudinal data assessed over 8 years for 2063 women using latent growth curve models revealed that high levels of discrimination and hostility predicted greater allostatic load, and high perceived stress predicted a more rapid rate of increase of allostatic load. African American women and women with low income and low education all experienced higher levels of allostatic load. These relationships were mediated by higher levels of discrimination, perceived stress, and hostility [26]. This approach to understanding the cumulative effects of stress can be applied to additional specific populations of women who experience high levels of stress.

## The thematic series

Unanswered questions about stress and midlife women will be addressed in this thematic series of *Women’s Midlife Health*. For example, what are the stressful life experiences that midlife women report most often, and which of these would be designated as most challenging? Are the same stressors identified by younger women, such as relationship issues, material resources, and occupational demands, also prevalent among midlife women? Of those challenges faced by midlife women, are there some that have the greatest negative impact in their lives? Which life events appear most salient to these women? Do they occur in clusters? For example, do life events manifest as co-occurring stressors when a woman divorces and is faced not only with a change in relationship with an adult partner, but also financial, housing, child-care, and possibly employment changes that are related to the divorce?

As seen in research on other portions of the lifespan, many of the instruments developed to study stress in men are not adequate for studying women. Investigators such as Norbeck [35] have developed measures to assess stressful life events in women of reproductive age. To date, however, no similar work has been done with a midlife population. Do we have data that would support modifying the commonly used instruments to assess life events among midlife women? Do existing instruments adequately capture the multiple transitions that women experience during midlife? Are they adequate for studies of unique populations of women? For example, in the U. S., African American women bear the results of a unique history of enslavement of their ancestors and policies that openly discriminated against them. What are the consequences for African American women’s health across the lifespan, and specifically during midlife? Studies of African American women have revealed experiences of racial discrimination, with what is now termed micro-aggressions exemplified by racist comments, news of racial injustices, and the intersection of sexism, ageism, and racism in employment situations [10, 11]. Geronimus and colleagues [36] wrote of the “weathering” effects (e.g., showing the morbidity and mortality patterns typical of much older white individuals) associated with living in a race-conscious society that stigmatizes and disadvantages Blacks. Using the U.S. NHANES data, they found that allostatic load was greatest among Black women, especially those 35-64 years of age, even when poverty was considered [36]. Data from SWAN have also shown how perceived discrimination is associated with an increased likelihood of negative health concerns [37, 38] and a reduction in positive health-related behaviors such screening for breast and cervical cancer [39].

We should also consider American Indian women, who have experienced a history marked by appropriation of their land, culture, and languages, and who currently grapple with stressors such as poverty and criminal violence. Among Mexican-American and other Hispanic women, some immigrants to the U.S. currently face threats of deportation.

A growing body of research about African American women suggests some unique responses to stress. For example, Woods-Giscombé [17] identified a profile of the “strong Black Woman,” proposing that the “Superwoman Schema,” influences both their experiences and reports of stress. Women’s descriptions of the Superwoman role included their sense of obligation to manifest strength and suppress emotions, resistance to being vulnerable or dependent, determination to succeed despite limited resources, and obligation to help others. Women participating in this study also identified contributing contextual factors, such as the historical legacy of racial or gender stereotyping or oppression, lessons from their foremothers, past history of disappointment, mistreatment, or abuse, and spiritual values. They indicated an array of perceived benefits, such as preservation of the self, and perceived liabilities, including the embodiment of stress [17].

Likewise, there is a need to understand the midlife experiences of lesbian, bisexual, and transsexual women [40]. Unique stressors may depend on how women identify themselves, their experience of attraction, and their behavior. Evidence suggests that, when the dimensions of identity, attraction, and behavior are not congruent, women experience greater stress and distress [41, 42].

In the context of midlife, it is important to consider the impact of stressful life experiences on women’s health, including both reproductive and cardiovascular systems. Do we understand the mechanisms by which physiological aspects of the stress response influence the HPO axis? What are the hormonal and HPA/HPO axis effects of stressful life events? And, ultimately, does poor health constitute an added stressor challenging midlife women’s coping efforts?

The papers in this thematic series examine stress at midlife in relation to a wide range of symptom experiences, in the context of family and negative life events, as associated with women’s work, and correlated with the challenges of violence and discrimination in women’s neighborhoods. The authors employ a range of stress measures, from the Perceived Stress Scale and Scale of Life Events to Epstein-Barr virus antibodies, C-reactive protein, and the circadian rhythms of catecholamines and cortisol. The study methods include cross-sectional research with single measures, cross-sectional research with multiple measures across a day, and longitudinal studies with multiple measures across many years. Some researchers focus singularly on midlife, while others consider the developmental origins of later life effects on ovarian reserve and cardiovascular disease. Cross-cultural differences in the report of stress are also considered.

## Conclusion

The purpose of this thematic series is to extend the conversation about stress to include women at midlife, and to examine where we are, and where we need to go, in order to better understand and provide relevant care for this growing population. A substantial number of women (though not all) are experiencing uncomfortable somatic, psychological, cognitive, and vasomotor symptoms at midlife [43, 44]. Substantial proportions are pulled in multiple directions by adolescent children and aging parents [28, 45]. A significant group of women at midlife cannot afford to retire and, therefore, must remain in difficult occupational situations, while others confront discrimination and violence in their everyday lives [26, 46].

There are many sources of stress, and the authors included in this thematic series aim to enhance our understanding of stress in relevant ways. They examine potential reproductive and health outcomes related to stress in order to answer pressing questions and to direct future research.
